# Assessment of Urinary Exosomal NHE3 as a Biomarker of Acute Kidney Injury

**DOI:** 10.3390/diagnostics12112634

**Published:** 2022-10-30

**Authors:** Yanting Yu, Zhiyun Ren, Anni Xie, Yutao Jia, Ying Xue, Ping Wang, Daxi Ji, Xiaoyan Wang

**Affiliations:** 1 Department of Nephrology, Nanjing BenQ Medical Center, The Affiliated BenQ Hospital of Nanjing Medical University, Nanjing 210019, China; 2 The Core Laboratory, Nanjing BenQ Medical Center, The Affiliated BenQ Hospital of Nanjing Medical University, Nanjing 210019, China

**Keywords:** acute kidney injury, exosome, NHE3, biomarkers

## Abstract

The diagnosis of acute kidney injury (AKI) traditionally depends on the serum creatinine (Scr) and urine output, which lack sufficient sensitivity and specificity. Using urinary exosomes as a biomarker has unique advantages. To assess whether urinary exosomal Na^+^/H^+^ exchanger isoform 3 (NHE3) protein could serve as a biomarker of AKI, we constructed four AKI rat models: cisplatin (7.5 mg/kg) injected intraperitoneally (IP), furosemide (20 mg/kg, IP) with a low-NaCl (0.03%) diet, a low-NaCl (0.03%) diet with candesartan (1 mg/kg, IP) and bilateral ischemia and reperfusion (I/R) injury for 40 min. Additionally, we assessed six sepsis-associated AKI patients and six healthy volunteers. Urinary exosomes were extracted by ultracentrifugation, and the NHE3 protein abundance was tested by immunoblotting for all the AKI rats and human subjects. The isolated cup-shaped particles with an average diameter of 70 nm and enrichment in CD63 were identified as exosomes. NHE3 abundance was six times higher in exosomes than in the whole urine. In cisplatin-induced AKI rats, urinary exosomal NHE3 was increased on day 2, one day earlier than the increases in Scr and blood urea nitrogen (BUN). In additional rats, urinary exosomal NHE3 decreased along with the decline in Scr after EPO pretreatment. In volume-depletion AKI induced by furosemide injection with a low-NaCl diet, the urinary exosomal NHE3 expression was higher than that in the control. Under a low-NaCl diet with candesartan-related AKI, the urinary exosomal NHE3 was elevated on day 5, earlier than Scr. In I/R-injury AKI, the urinary exosomal NHE3 was also raised compared with that in the control. In humans, the urinary exosomal NHE3 level was also elevated in sepsis-associated AKI patients in comparison with that in the healthy volunteers. The urinary exosomal NHE3 was increased in multiple AKI; it may be used as a diagnostic biomarker of AKI.

## 1. Introduction

Acute kidney injury (AKI) is a global public health concern associated with high morbidity [1] and mortality [2]. The lack of recognition of AKI delays diagnoses. Non-recognized AKI delays the diagnosis. A survey of 2.2 million patients showed that 74.2% of patients with identifiable AKI did not have their disease recognized by physicians, and 17.6% of recognized AKI cases were given delayed diagnoses [3]. The high rate of non-recognition is due to a lack of alarming symptoms or specific early biomarkers. AKI is defined by a fixed percentage increase in serum creatinine (Scr), decrease in urine output, or both within 7 days, according to the Kidney Disease: Improving Global Outcomes (KDIGO) criteria [4]. Scr has poor sensitivity and specificity for detecting or grading the severity of AKI, and lags behind both renal injury and renal recovery. Furthermore, urine output is difficult to quantify precisely without an indwelling catheter. Hence, novel serum or urinary biomarkers for AKI are necessary and have been a hot topic in the field.

Several urinary biomarkers have been discovered as noninvasive indicators for the prediction and diagnosis of AKI, including kidney injury molecule-1, N-acetyl-beta-D-glucosaminidase [5,6], neutrophil gelatinase-associated lipocalin [7] and liver fatty acid-binding protein [8]. The urinary product of tissue inhibitor metalloproteinase and insulin growth factor binding protein-7 [9] and urinary thioredoxin [10] are also clinically available. However, each biomarker currently has limitations. Moreover, their adoption into routine clinical care has been slow, probably due to the cost, availability of testing platforms, variability in assay techniques and results, and lack of governance approval. In order to improve the early detection of AKI, additional urinary biomarkers need to be identified.

Urinary exosomes containing apical membranes and intracellular fluid are normally secreted into the urine from all the nephron segments, and may carry biomarkers, such as proteins and microRNA, of renal dysfunction and structural injury. The application of urinary exosomes as markers has benefited from the extraction of exosomes from healthy human urine by Pisitkun Trairak and colleagues [11]. They first extracted urinal exosomes and identified 295 unique proteins, including several transporters and channels mainly associated with the apical membrane. Na^+^/H^+^ exchanger isoform 3 (NHE3) is expressed in the apical membrane of the mammalian proximal convoluted tubule and thick ascending limb [12]. NHE3 belongs to the mammalian NHE protein family and catalyzes the electroneutral exchange of extracellular sodium for intracellular protons across cellular membranes. In fact, all the major sodium transporters expressed along the nephron, including NHE3, the Na–K–2Cl cotransporter, and the thiazide-sensitive Na–Cl cotransporter, are detectible in the urine of rats by means of antipeptide antibodies, suggesting that profiling sodium transporters in urine may become a useful test tool for detecting and classifying kidney diseases [13]. The detection of urinary exosomal proteins [14] and microRNAs [15] in kidney disease allows experts to explore the possibility of exosomes serving as specific biomarkers for AKI, since they are actively secreted by live cells. In this study, we detected the urinary exosomal NHE3 protein in various AKI rats and sepsis-associated AKI patients, aiming to assess its potential as a new early biomarker for AKI.

## 2. Materials and Methods

### 2.1. Urine Collection and Exosomal Extract

Twenty-four-hour urine from rats and morning urine from patients were centrifuged at 4000× *g* for 10 min to remove debris and stored in a −80 °C freezer. The exosomes were isolated by ultracentrifugation according to the manufacturer’s instructions [11]. The urine samples were centrifuged at 17,000 g for 10 min at 4 °C. The supernatant was collected as supernatant 1, and the precipitation with 2 mL of isolation buffer containing 200 mg/mL DTT was centrifugated at 17,000 g for 10 min again. The second supernatant was collected as supernatant 2. Supernatants 1 and 2 were isolated by centrifugation at 200,000× *g* for 1 h (h). The pellets were resuspended in 60 uL of isolation solution containing 1 g/mL leupeptin and 0.1 mg/mL PMSF. The isolated exosomes were kept at 2–8 °C for the next experiments.

### 2.2. Animals and Experimental Protocol

#### 2.2.1. Animal

Eight-week-old male Sprague-Dawley (SD) rats weighing 300–350 g were purchased (Beijing, China) and were used for various studies. The animal experiments were approved by the Committee on Animal Care of Nanjing Medical University and conducted according to the National Institutes of Health Guidelines for the Care and Use of Laboratory Animals. All the studies involving animals are reported in accordance with the ARRIVE (Animal Research: Reporting of In Vivo Experiments) guidelines. The animals were maintained under 12 h light/dark cycle in a temperature- and humidity-controlled facility and were allowed a minimum of 7 days for acclimation. The rats were placed in individual metabolic cages (Shanghai Yuyan Instruments, Ltd., Shanghai, China), allowing daily ration feeding and the collection of urine. Blood and tissue samples were harvested at the end time and processed for various studies.

#### 2.2.2. Cisplatin-Induced AKI

Twelve SD rats were divided into a control group and cisplatin group. Cisplatin (Cis, 7.5 mg/kg; Sigma-Aldrich, Darmstadt, Germany) was administered in a single intraperitoneal injection, as described in previous research [16], and the Cis-group rats were fed for 1 day, 2 days, or 3 days. The control rats received the vehicle, 0.9% saline, and were sacrificed on day 3 (*n* = 3 per group). For the rescue experiment, 13 SD rats were randomly assigned into the control (*n* = 4), cisplatin (Cis, *n* = 4) and cisplatin-with-erythropoietin (Cis + EPO, *n* = 5) group. EPO (5000 U/kg; EPIAO) was administered 2 times, 15 min before cisplatin and 2 days after cisplatin administration. All the rats were sacrificed on day 3.

#### 2.2.3. Volume-Depletion-Induced AKI

Eight SD rats were divided randomly into a control group and a volume-depletion (VD) group according to a previous method [17]. The VD-group rats were fed food with 0.4% NaCl (Jiangsu Xie Tong Pharmaceutical Bioengineering, Ltd., Nanjing, China) for 18 h (−18 h) and then were injected intraperitoneally with furosemide (BP547, Sigma, 20 mg/kg) once (0 h), 8 h later (8 h) receiving the second furosemide injection at the same dose, and were sacrificed at 30 h. Throughout the 48 h, the rats were fed food containing 0.4% NaCl. The control rats were administered a low-salt (0.03% NaCl) diet, and the other procedure was the same. Blood was collected at 30 h. Urine samples for the control group were collected throughout the 48 h. Urine from the VD group was collected at different time points: −18 h to 0 h, 0–8 h, 8–24 h, and 24–30 h.

#### 2.2.4. Low-NaCl Diet with Candesartan-Related AKI

All 12 SD rats were randomly divided into 2 groups. The low-NaCl-group rats were fed food with 0.03% NaCl. The normal-salt group was fed food with 0.8%NaCl. All the rats were intraperitoneally injected with angiotensin receptor blocker (ARB), candesartan (1 mg/kg/day, PHR1854, Sigma-Aldrich, Darmstadt, Germany) for one week. The rats were sacrificed on day 7. The low-NaCl-diet-with-candesartan model was established previously to study epithelial sodium channel expression in the kidney [18].

#### 2.2.5. Ischemia/Reperfusion-Related AKI

SD rats were assigned randomly to 2 groups: a sham-operation group (control) and I/R group (*n* = 6/group). The rats were placed under surgical anesthesia with 0.5 mg/kg chloral hydrate (Macklin, Shanghai, China). The abdomen was opened, the left renal pedicles were located for clamping, and then the right, and each was clamped for 40 min at 37 °C [17]. After releasing the cross-clamps, the kidneys were re-perfused and the color returned to the original. The abdomen was closed in two layers. The sham-operated group was subjected to a similar surgical procedure, except for the clamp.

### 2.3. AKI Patients

A total of 12 subjects were enrolled at the Affiliated Benq hospital of Nanjing Medical University between January 2022 and June 2022. Six participants were diagnosed with AKI based on the criteria of KDIGO [4]. The other 6 participants from the Health Management Center served as healthy controls. The etiology of AKI was clinically considered to be septic shock according to the latest international consensus. In brief, sepsis is defined as life-threatening organ dysfunction caused by a dysregulated host response to infection. Septic shock is a subset of sepsis in which the underlying circulatory and cellular/metabolic abnormalities are profound enough to substantially increase mortality [19]. The exclusion criteria were patients younger than 18 or older than 80 years; using angiotensin-converting-enzyme inhibitors, angiotensin-receptor blockers, or diuretics within the previous few weeks; and a urinary tract infection. Morning urine samples, blood samples, and blood pressures of all the subjects were collected.

### 2.4. Transmission Electron Microscopy

The pellet obtained after centrifuging the urine at 200,000 g was rinsed and post-fixed in 1% osmium tetroxide. The samples were embedded in 10% gelatin, and fixed and cut into several blocks (<1 mm^3^). The samples were dehydrated in increasing concentrations of alcohol and infiltrated with increasing concentrations of Quetol-812 epoxy resin mixed with propylene oxide. The samples were embedded in pure fresh Quetol-812 epoxy resin and polymerized. Ultrathin sections (100 nm) were cut by using a Leica UC6 ultramicrotome and post-stained with uranyl acetate for 10 min and lead citrate for 5 min at room temperature, followed by observation with a transmission electron microscope (HT-7700, Hitachi, Ltd., Tokyo, Japan) operated at 100 kV.

### 2.5. Nanoparticle Tracking Analysis

The 200,000 g pellets were sized and enumerated by nanoparticle tracking analysis (NTA) using a Nano FCM (N30 E) instrument. NTA is a light-scattering technique that uses video analysis for the sizing and enumeration of extracellular vesicles. Urine samples were collected and diluted in PBS to a particle concentration within the range of 2–10 × 10^8^/mL (the optimal working range of the system). An approximately 100 μL diluted sample was loaded into the sample chamber, and 60 s videos were recorded for each sample with a shutter speed of approximately 30 ms and a camera gain between 250 and 650. The settings for software analysis were as follows: detection threshold, 30–50; blur, 535; and minimum expected particle size, auto. The size distributions are presented as 5–6 video recordings per sample.

### 2.6. Biochemical Analyses

For rats, the Scr was measured with the QuantiChrom Creatinine Assay Kit (cat.: DICT-500, Bioassay Systems, Hayward, CA, USA), and BUN was measured with the QuantiChrom Urea Assay Kit (cat.: DIUR-500, Bioassay Systems) according to the manufacturer’s instructions. The patient’s serum creatinine and BUN were tested in the Clinical Laboratory using a Roche instrument (cobas8000).

### 2.7. Western Blotting

The different fractions of urine from rats separated by differential centrifugation were mixed with 1:4 vol/vol of Laemmli buffer and heated to 60 °C for 15 min. The loading volume for immunoblotting was normalized to the urine creatinine content. Kidney homogenates were prepared on ice using RIPA lysis buffer. The total protein concentration was measured with a BCA kit (P0009, Beyotime). Equal amounts of protein from each sample were electrophoresed on SDS–polyacrylamide gels and electrotransferred onto nitrocellulose membranes, which were incubated with the primary antibodies anti-CD63 (25682-1-AP, Proteinch, Wuhan, China), anti-NHE3 (sc58636, Santa Cruz Biotechnology, Inc., Dallas, TX, USA) and anti-β-actin (A2066, Sigma-Aldrich, Darmstadt, Germany). An X-ray film was exposed to the chemoluminal signals from HRP-conjugated secondary antibodies (1:10,000). The band densities of the proteins were quantified using the Image J software and are expressed as percentages of the relevant control density.

### 2.8. Histology Examination

Renal tissues were fixed in 10% paraformaldehyde and embedded in paraffin, and 3μm-thick sections were processed for periodic acid-Schiff (PAS) staining using standard methods. The tubular injury score was quantified by counting the percentage of tubule dilation, loss of brush border, cast formation, and cell necrosis as follows: 0, none; 1, <10%; 2, 11–25%; 3, 26–45%; 4, 46–75%; and 5, >76% in 10 HPFs [20]. The histopathological evaluation and grading of the tissue samples were conducted blindly by two experienced pathologists. The tissue sections were viewed with a Nikon Eclipse 80i microscope equipped with a digital camera (DS-Ri1, Nikon, Shanghai, China).

### 2.9. Immunofluorescent Staining

Kidney cryosections at a 3 μm thickness were fixed for 15 min in 10% paraformaldehyde, followed by permeabilization with 0.2% Triton X-100 in phosphate-buffered saline (PBS) for 5 min at room temperature. After blocking with 2% donkey serum for 60 min, the slides were immunostained with anti-NHE3 (sc58636, Santa Cruz Biotechnology, Inc., Dallas, TX, USA) and, subsequently, with the appropriate fluorophore-conjugated secondary antibodies (1:500; Molecular Probes, Grand Island, NY, USA). The slide was mounted using ProLong Gold Antifade Mountant (Thermo Fisher Scientific, Inc., Waltham, MA, USA) and imaged with an LSM 800 confocal microscope (Carl Zeiss, Oberkochen, Germany).

### 2.10. Statistical Analyses

All the quantitative data are expressed as the mean ± SEM. Statistical analysis of the data was performed using the SPSS 22.0 software (SPSS, Chicago, IL, USA). A comparison between groups was made using one-way ANOVA, followed by post hoc SNK or post hoc LSD. For comparison between two groups, Student’s *t*-test was used, and paired *t*-tests were used for group comparisons. *p*-values less than 0.05 were considered significant.

## 3. Results

### 3.1. Isolation and Identification of Urinary Exosome and Urinary NHE3 Protein

**Figure 1:**
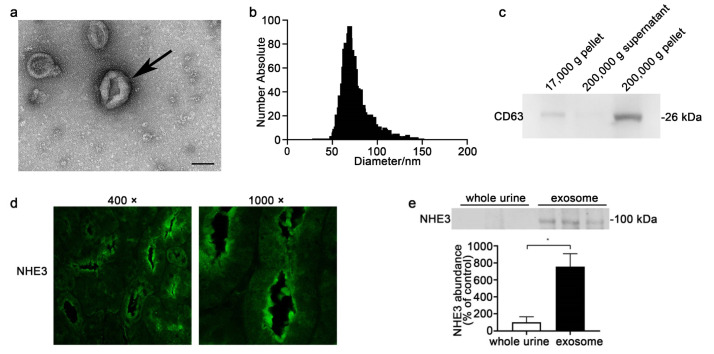
Characterization of urinary exosomes and NHE3 protein expression in kidney and urine. (**a**) Electron micrograph image of urinary exosomes. The urine was centrifuged at 17,000× *g* for 10 min, and the supernatant was ultracentrifuged at 200,000× *g* for 1 h to pellet the exosomes. The arrowhead shows round, cup-shaped vesicles. Scale bar = 100 nm. (**b**) Particle size distribution in purified pellets is consistent with the size range of exosomes (average size: around 75 nm), as measured by using a Zeta View^®^ particle tracking analyzer. (**c**) Detection of CD63 protein in different urinary fractions from the same urine sample by Western blot analysis for Sprague-Dawley (SD) rats. (**d**) Immunofluorescent staining images for NHE3 in the kidneys of normal SD rats. (**e**) NHE3 protein expression in the whole urine and urinary exosomes from normal SD rats. The loading amount was corrected according to the urinary creatinine content, and the same amount of creatinine was loaded. * *p* < 0.05.

We extracted exosomes by ultracentrifugation. The 200,000 g pellets were characterized using a transmission electron microscope, a nano-tracking analyzer, and Western blotting. The electron microscopy showed that the pellets were round and cup-shaped, with a diameter of less than 100 nm (Figure 1a). The nano-tracking analyzer revealed that the diameter of the particles was 50–100 nm, with an average of 75 nm (Figure 1b). Western blotting showed that the CD63 protein, a marker of exosomes, had the highest expression in the 200,000 g precipitate and a small amount in the 17,000 g precipitate, but almost no expression in the 200,000 g supernatant (Figure 1c). The immunofluorescent staining for NHE3 (apical membrane) reflected NHE3’s location in the kidney, mainly at proximal tubules (Figure 1d). Next, we compared the NHE3 levels in the whole urine and urinary exosomes. The NHE3 protein abundance in the exosomes was six times higher than that in the whole urine (755.6 ± 152.6 vs. 100 ± 63.5, *p* < 0.05) (Figure 1e).

### 3.2. Urinary Exosomal NHE3 Expression in Cisplatin-Induced AKI

**Figure 2:**
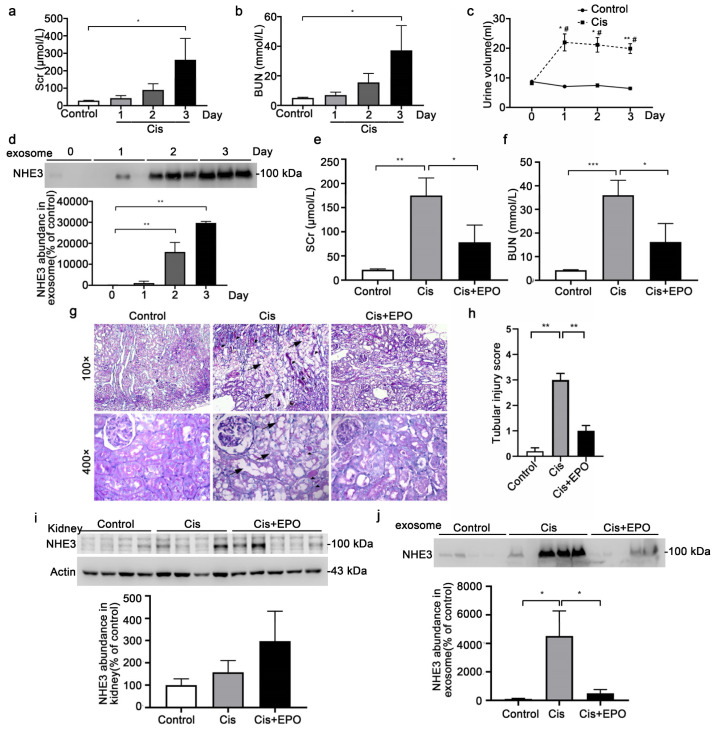
NHE3 expression in the cisplatin-induced acute kidney injury rats. (**a**,**b**) Comparison of Scr and BUN between the control and cisplatin-treated rats. Twelve rats were intraperitoneally injected with saline (control) or cisplatin (7.5 mg/kg; Sigma) and were sacrificed at 1 day, 2 days, or 3 days (*n* = 3 per group). (**c**) The daily urine outputs of the control and cisplatin rats. * vs. control group. ^#^ vs. day 0. (**d**) Urinary exosomal NHE3 protein expression for control rats and cisplatin-treated rats at day 1, day 2 and day 3. (**e**,**f**) Comparison of serum creatine and BUN levels among the control (*n* = 4), cisplatin (Cis, *n* = 4) and cisplatin + EPO (Cis + EPO, *n* = 5) groups. Cisplatin (7.5 mg/kg) was administered on day 1 intraperitoneally. EPO (5000 U/kg; EPIAO) was administered 2 times, 15 min before cisplatin administration and 2 days after cisplatin administration. All the rats were sacrificed on day 3. (**g**) Renal histological changes in the different groups. Kidney sections were stained with PAS to assess morphological changes. Arrowheads indicate tubule dilation, loss of brush border or epithelial cell necrosis. Triangles show cast formation. (**h**) Renal tubular injury score. (**i**) NHE3 protein level in the kidney. (**j**) Urinary exosomal NHE3 protein expression among the three groups. The data are presented as the mean ± SE. * *p* < 0.05, ** *p* < 0.01, *** *p* < 0.001. ^#^ *p* < 0.05. Scr, serum creatinine; BUN, blood urea nitrogen.

The Scr (379.7 ± 32.9 vs. 28.7 ± 1.8 μmol/L, *p* < 0.01) and BUN (53.9 ± 1.9 vs. 5.0 ± 0.4 mmol/L, *p* < 0.01) were markedly increased at day 3 after cisplatin injection (Figure 2a,b). The 24 h urine output was much higher in the cisplatin group, whether compared with the control or the output in the cisplatin group at day 0, which was consistent with a previous study [21] (Figure 2c). The urinary exosomal NHE3 increased on day 2 (15, 880 ± 2866 vs. 100 ± 44.3, *p* < 0.01), 1 day before Scr increased, and continued to increase on day 3 (29,688 ± 475 vs. 100 ± 44.3, *p* < 0.01). Next, we constructed an AKI recovery model by pretreatment with EPO 15 min before cisplatin injection and 2 days after cisplatin injection. After EPO pretreatment, the increases in Scr and BUN were reversed (78.2 ± 35.4 vs. 175 ± 36.3 μmol/L, *p* < 0.05) (16.2 ± 7.8 vs. 35.9 ± 6.3, *p* < 0.05), respectively (Figure 2e,f). Histology showed that the tubule dilation, loss of brush border and cast formation were ameliorated (Figure 2g). The tubular injury score was significantly increased in the cisplatin group and improved after EPO pretreatment (Figure 2h). There was no difference in the NHE3 protein abundance in the kidney among the three groups (Figure 2i). The urinary exosomal NHE3 was increased in the cisplatin group (4517 ± 1758 vs. 100 ± 41.63, *p* < 0.05) and decreased after EPO pretreatment (501.2 ± 253.1 vs. 4517 ± 1758, *p* < 0.05), reaching the level of the control group (Figure 2j).

### 3.3. Urinary Exosomal NHE3 Level in Volume-Depletion-Induced AKI

**Figure 3:**
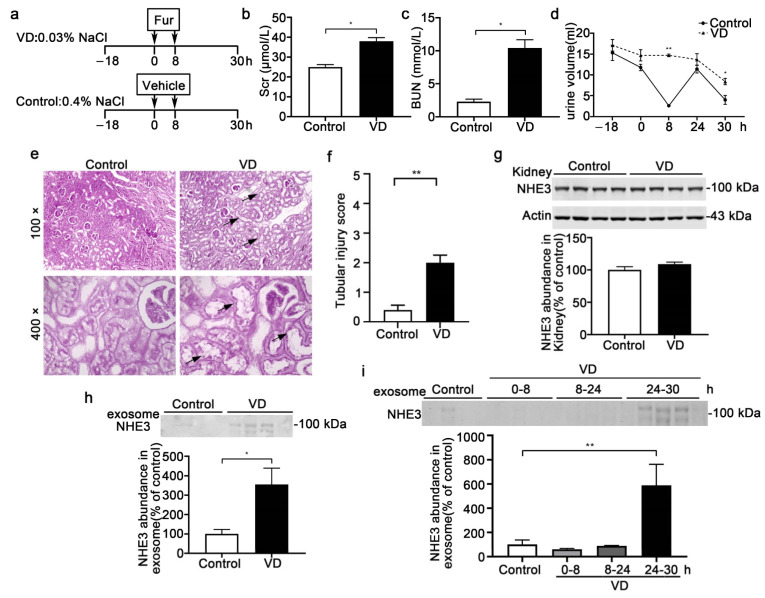
The NHE3 expression in the volume-depletion (VD)-induced AKI model. (**a**) The steps of the VD-AKI model. The VD-group rats were fed food with 0.4% NaCl from −18 h to 30 h and intraperitoneally injected with furosemide (20 mg/kg) two times at 0 h and 8 h, while the control group was fed with 0.03% NaCl food and injected with the vehicle at the same time points (*n* = 4/group). (**b**–**d**) Renal-function indicators-the serum creatinine levels (**b**), BUN (**c**) and urine volume (**d**)—were measured. (**e**) Representative micrographs of histological analysis (PAS staining) of renal tissue from control and VD-AKI rats. Arrowheads indicate tubule dilation or loss of brush border. (**f**) Renal tubular injury score. (**g**) Immunoblot analyses and quantification of NHE3 protein levels in kidneys. (**h**) Urinary exosomal NHE3 protein expression in control (all for 48 h urine) and VD-AKI rats (24–30 h). (**i**) Urinary exosomal NHE3 in VD rats in different periods: –18–0 h, 0–8 h, 8–24 h and 24–30 h. The data are presented as the means ± SEs (*n* = 4/group). * *p* < 0.05 and ** *p* < 0.01 vs. the vehicle control group. Fur: furosemide; h: hour.

A low-NaCl intake and furosemide injection is recognized as a volume-depletion (VD) [17] AKI model (Figure 3a). The increases in Scr (38 ± 1.8 vs. 25 ± 1.2 μmol/L, *p* < 0.05) and BUN (10.4 ± 1.2 vs. 2.3 ± 0.4 mmol/L, *p* < 0.05) mean that the VD-AKI model was successfully established (Figure 3b,c). The elevation of urine output after furosemide treatment was in accordance with volume depletion (Figure 3d). The pathological injury in VD-AKI included glomerular capillary loop shrinkage and tubule dilation (Figure 3e). The tubular injury score was higher in the VD group (Figure 3f). We detected the abundance of the NHE3 protein in the kidney, there was no change between the control and VD groups (Figure 3g). Otherwise, the exosomal NHE3 in all the 30 h urine samples was increased in the VD group (589.3 ± 173 vs. 100.3 ± 36.8, *p* < 0.05) (Figure 3h). To detect whether it could serve as an early biomarker, we split all the urine into different periods: 0–8 h, 8–24 h and 24–30 h urine. The urinary exosomal NHE3 was increased at the end time of 24–30 h (355.5 ± 83.9 vs. 100 ± 22.6, *p* < 0.01) compared with the control. However, it did not change at either 0–8 h or 8–24 h compared with the control (Figure 3i).

### 3.4. Urinary Exosomal NHE3 Excretion under AKI Induced by Low-NaCl Intake and Candesartan

**Figure 4:**
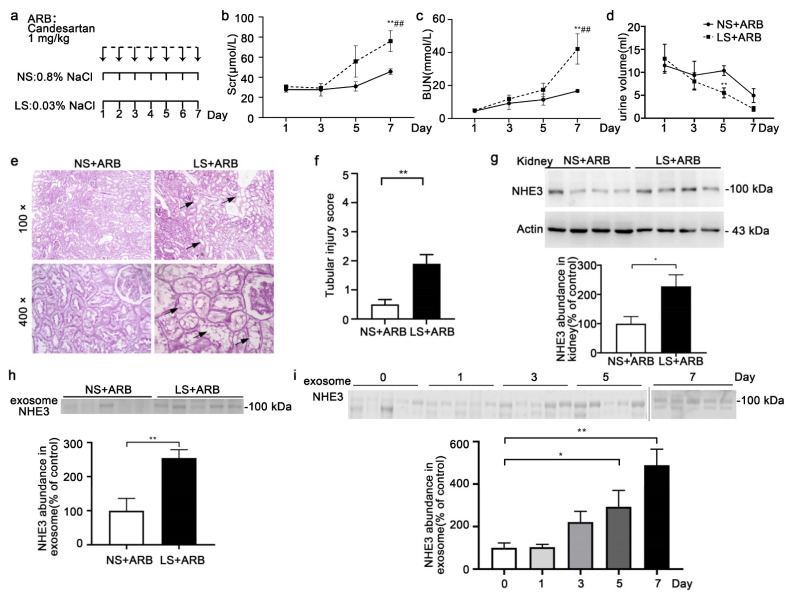
The NHE3 expression in rats with AKI induced by a low-NaCl diet and candesartan. (**a**) Method of inducing AKI with a low-NaCl diet and candesartan. SD rats were intraperitoneally injected with candesartan (1 mg/kg/day), an ARB, for one week and fed food with low salt with a NaCl content of 0.03% (LS + ARB) or normal salt with a NaCl content of 0.8% (NS + ARB). All the rats were sacrificed on the 7th day. (**b**–**d**) Scr (**b**), BUN (**c**) and urine volume (**d**) on days 1, 3, 5, and 7 between two group rats. * vs. NS + ARB, ^#^ vs. day 1. (**e**) Histological analysis (PAS staining) of renal tissue from NS + ARB and LS + ARB rats. Arrows indicate renal tubular foam cells. Arrowheads indicate tubule dilation. (**f**) Renal tubular injury score. (**g**,**h**) Immunoblot analyses and quantification of the NHE3 protein in the kidney (**f**) and urinary exosomes (**g**) on day 7 for the NS + ARB and LS + ARB rats. (**i**) Urinary exosomal NHE3 expression on days 0, 1, 3, 5 and 7 for LS + ARB-induced AKI rats. The data are presented as the means ± SEs (*n* = 4–5). * *p* < 0.05 and ** *p* < 0.01. ^##^ *p* < 0.01.

A low-salt intake with the administration of candesartan can induce AKI, whether in normotensive, hypertensive, or nephritic rats, as reported in our other article (under review). The SD rats were fed a 0.03% NaCl or 0.8% NaCl diet and intraperitoneally injected with candesartan, an angiotensin receptor blocker (ARB), for 7 days (Figure 4a). Both the Scr (76 ± 10.3 vs. 45.7 ± 2.7 μmol/L, *p* < 0.05) and BUN (42.2 ± 9.1 vs. 16.7 ± 0.9, *p* < 0.05) increased gradually, with a statistically significant difference on day 7 between the two groups (Figure 4b,c). The urine output decreased gradually in both groups (Figure 4d). Histological analysis of renal tissue showed tubule dilation and foam cells gathering, indicating tubular injury (Figure 4e,f). The NHE3 protein in the kidney tissue (228.1 ± 39.3 vs. 100 ± 24.2, *p* < 0.05) was increased in the LS + ARB group compared with the NS + ARB group (Figure 4g). The urinary exosomal NHE3 protein (254.8 ± 24.5 vs. 100± 36.1, *p* < 0.01) was markedly increased, and the trend was similar to that in the kidney tissue (Figure 4g, h). We present the time course of urinary exosomal NHE3 expression on days 0, 1, 3, 5, and 7 in LS + ARB-induced AKI rats. It gradually increased, with a statistically significant difference on day 5 (293 ± 7.2 vs. 100 ± 22.8, *p* < 0.05), which was 2 days earlier than the Scr increase, and it was still elevated at day 7 (702.8 ± 108.1 vs. 100 ± 22.8, *p* < 0.01) (Figure 4i).

### 3.5. Urinary Exosomal NHE3 Level in I/R AKI

**Figure 5:**
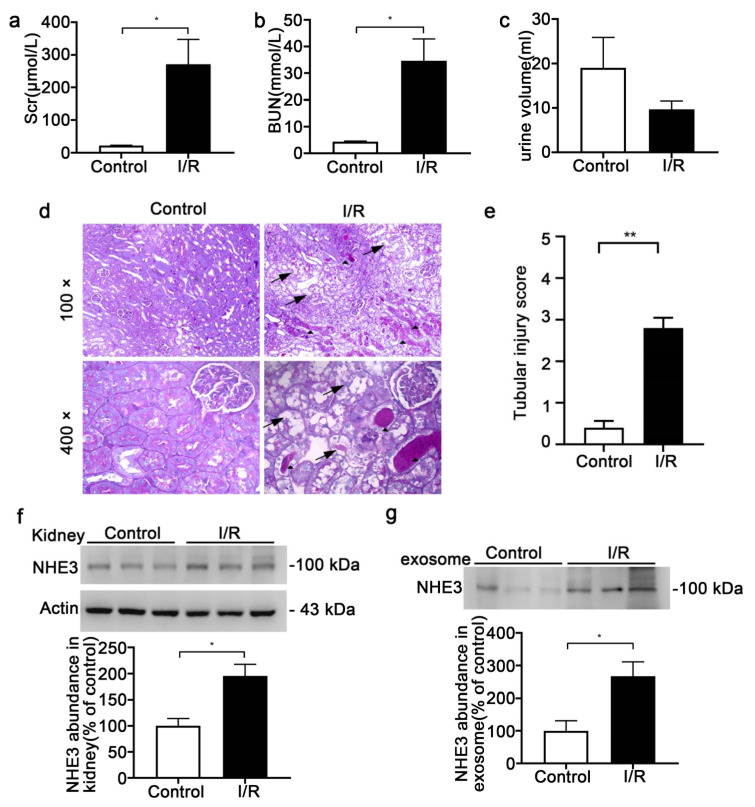
The NHE3 expression in the ischemia/reperfusion (I/R)-induced AKI rats. (**a**–**c**) Changes in Scr (**a**) and BUN (**b**) and urine output (**c**) from control or I/R SD rats with bilateral ischemia for 40 min, and reperfusion for 24 h. (**d**) Representative micrographs of histological analysis (PAS staining) of renal tissue from control or I/R rats. Arrowheads indicate tubule dilation, loss of brush border or epithelial cell necrosis. Triangles show cast formation. (**e**) Renal tubular injury score. (**f**) Immunoblot analyses and quantification of NHE3 from kidney tissue. (**g**) Urinary exosomal NHE3 protein levels for the control and I/R rats as analyzed by immunoblotting. The data are presented as the means ± SEs (*n* = 3). * *p* < 0.05 and ** *p* < 0.01 vs. the vehicle control group.

The rats were bilaterally clamped for 40 min and reperfused for 24 h. The Scr (270.8 ± 76.5 vs. 22 ± 1.0 μmol/L, *p* < 0.05) and BUN (34.7 ± 8.2 vs. 4.3 ± 0.2 mmol/L, *p* < 0.05) were prominently elevated (Figure 5a,b). The urine volume showed a slight decline but showed no statistically significant difference (Figure 5c). The kidney histomorphology showed tubular dilation, brush border loss and cast formation in the I/R group, and the tubular injury score was higher (Figure 5d,e). Both the kidney NHE3 (195.2 ± 22.6 vs. 100 ± 14.1, *p* < 0.05) and urinary exosomal NHE3 (267 ± 44.4 vs. 100 ± 31.1, *p* < 0.05) were raised in the I/R rats compared with the control rats (Figure 5f,g).

### 3.6. Urinary Exosomal NHE3 Abundance in Sepsis-Associated AKI Patients

**Figure 6:**
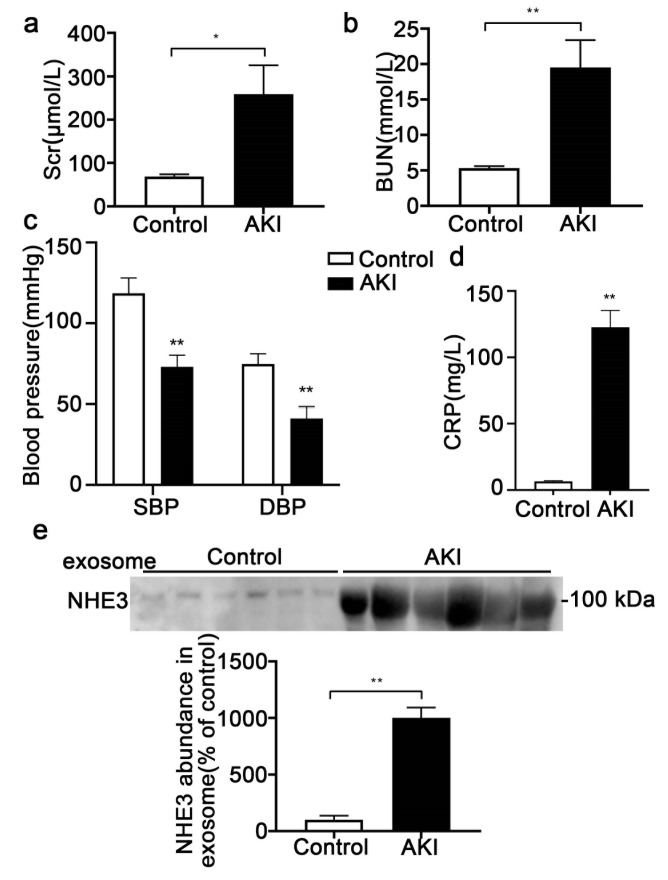
Urinary exosomal NHE3 expression in sepsis-associated AKI patients. (**a**, **b**) Scr and BUN of sepsis-associated AKI patients and healthy volunteers were measured (*n* = 6). (**c**) Systolic blood pressure and diastolic blood pressure of sepsis-associated AKI patients and healthy volunteers. (**d**) CRP levels in the two groups of subjects. (**e**) Immunoblot analyses and quantification of NHE3 from morning urinary exosomes. Each value was normalized to the urine creatinine. The data are presented as the means ± SEs. * *p* < 0.05 and ** *p* < 0.01. SBP, systolic blood pressure; DBP, diastolic blood pressure; CRP, C reactive protein.

Six sepsis-associated AKI patients and six healthy volunteers were enrolled. Among the six AKI patients, four were diagnosed with pneumonia, one with cholecystitis, and one with gastroenteritis. There were no urinary tract infections. Two patients were administered levofloxacin, two cefoperazone sulbactam, one piperacillin tazobactam and two ornidazole. None had urinary tract infections. None received aminoglycosides, iodinated contrast media, or any diuretics that may have damaged renal tubules and affected the expression of NHE3. The age (55 ± 7.2 vs. 58 ± 6.5, *p* > 0.05) and gender (male%, 50% vs. 50%, *p* > 0.05) had no differences between the AKI and control groups. The Scr (258.5 ± 66.9 vs. 68.7 ± 5.4 umol/L, *p* < 0.05) (Figure 6a) and BUN (19.5 ± 3.9 vs. 5.3 ± 0.3 mmol/L, *p* < 0.01) were significantly elevated (Figure 6b). The systolic blood pressure (73 ± 2.9 vs. 118.5 ± 3.9 mmHg, *p* < 0.01) and diastolic blood pressure (41 ± 3.1 vs. 74.8 ± 2.6 mmHg, *p* < 0.01) of the AKI patients were both lower (Figure 6c), and the serum C reactive protein (CRP), a marker of infection, was markedly increased (122.7 ± 12.6 vs. 6.6 ± 0.6 mg/L, *p* < 0.01) compared to those in the healthy volunteers (Figure 6d), indicating that septic shock occurred. The NHE3 in urinary exosomes (1003 ± 89.7 vs. 100 ± 36, *p* < 0.01) was also significantly increased compared with that in the healthy control group (Figure 6e).

## 4. Discussion

In the present study, we found that the urinary exosomal NHE3 protein was increased in rats with AKI induced by cisplatin, volume depletion, I/R injury, and low NaCl with candesartan, and also elevated in sepsis-associated AKI patients. Furthermore, the urinary exosomal NHE3 was detected 1 day earlier than Scr in cisplatin-induced AKI rats, and 2 days earlier in AKI induced by low NaCl with candesartan. This indicated that urinary exosomal NHE3 can be used as a noninvasive diagnostic marker of various AKIs, and even as an early marker for some types of AKI.

Exosomes are defined as vesicles ~40 to 160 nm (average: ~100 nm) in diameter that are secreted when multivesicular bodies fuse with the plasma membrane [22]. Exosomes are found in all biological fluids and are secreted by all cells. Depending on the cell of origin, exosomes contain many constituents of a cell, including DNA, RNA, lipids, metabolites, and cytosolic and cell-surface proteins. Membrane proteins such as transporters and ion channels are expected to be highly enriched in exosomes [23]. Exosome detection in biological fluids potentially offers a multicomponent diagnostic readout [24]. Urinary exosomes are a rich source of biomarkers because they are released from every segment of the nephron [25]. In 2004, Pisitkun Trairak [11] and colleagues isolated and identified a series of exosomal proteins from healthy human urine; the research into urinary exosomes as biomarkers has since risen rapidly. Exosomal fetuin-A was identified by proteomics as a novel urinary biomarker for detecting AKI [17], and the transcription factor ATF3 might serve as a biomarker of renal tubular cell injury [14]. Urinary exosomal CD26 [26] was associated with renal reversal and recovery from AKI. NHE3 was the most abundant sodium transporter in renal tubules, localized in the apical membrane and subapical endosomes of renal proximal tubular cells and in the apical membranes of thick ascending limb cells. A few studies have focused on the urinary exosomal NHE3 [13], but these studies had some shortcomings, such as limited sample sizes or complex clinical backgrounds.

In our study, urinary exosomal NHE3 could be detected in normal rats (Figure 1e) and healthy human subjects (Figure 6e), although the amount was very small, inconsistent with a previous study, in which NHE3 was not detected in urine from controls. The reason may be related to the difference in the way we acquired exosomes. We had one more step than the former, centrifuged at 17,000× *g* for 10 min, and then added the reducing agent DTT and repeated the centrifugation. This is an improved method of exosome extraction using ultracentrifugation developed in recent years [11]; hence, we may have obtained more exosomes. Previous studies observed that the levels of urinary NHE3 were increased in patients with prerenal azotemia, being six times as high in patients with acute tubular necrosis (ATN), but not in intrinsic acute renal failure (ARF) other than ATN. However, in clinical practice, prerenal azotemia (also named prerenal ARF) and ATN (representative of renal ARF) may co-exist, and it is difficult to distinguish ATN and intrinsic ARF without a renal biopsy. Therefore, our study detected urinary exosomal NHE3 from various AKI animal models of different etiologies, considering that the animal models were simple and the mechanism was clear. Moreover, in our study, the histopathology of the animal model can directly show the damage to renal tubules, which is complementary to previous studies.

The reason for the increase in urinary exosomal NHE3 was worth exploring. In the present four AKI models, the kidney NHE3 protein abundance was elevated in AKI induced by I/R and low NaCl with candesartan, but not in cisplatin- or volume-depletion-induced AKI. Hence, the cause could not be simply attributed to the elevation in renal tissue. Polyuria occurred in volume-depletion-induced AKI and cisplatin-induced AKI, which was associated with a reduction in medullary hypertonicity, due to qualitative changes in urea transporter proteins [21]. Though polyuria might cause the elevation of total urinary NHE3, our exosome loading volume was normalized to the urinary creatinine concentration. Since urinary exosomes are extracellular microvesicles that are actively secreted by living cells [27], an increase in urinary exosomal NHE3 is considered to indicate a stress response of the proximal tubular epithelial cells to medicines, volume changes, or other factors. Additionally, NHE3, as the most abundant sodium transporter in the renal tubules, which was localized in the apical membranes and subapical endosomes of renal proximal tubular cells and in the apical membranes of thick ascending limb cells, may also appear in the urine as a result of incomplete proximal tubule processing in proteinuria states (a form of overflow proteinuria) or be released during tubular cell apoptosis.

The cause and pathogenesis of AKI are complex, involving ischemia, sepsis, drug toxicity, and trauma. Cisplatin [28], ischemia and reperfusion [29], lipopolysaccharide [30], and volume depletion [17] have been widely used for AKI animal models. Furthermore, multiple AKI phenotypes exist clinically, and more than one phenotype may exist within the same individual. A low-NaCl diet with candesartan was used to study another sodium channel, ENaC, as early as 2005 [18], but kidney function was not observed. Proteinuria or hypertensive patients often take angiotensin II-receptor blockers and adopt low NaCl intakes clinically. We observed that the kidney function was damaged in SD rats or spontaneously hypertensive rats; then, we established a new AKI model using a low-NaCl diet with candesartan injection and explored the mechanism. A low NaCl intake with candesartan might promote the production of nitric oxide and lead to hypotension, ultimately inducing AKI (the data were unpublished). In this experiment, we detected the urinary exosomal NHE3 in this new AKI model, which was increased 2 days earlier than the Scr increase. The urinary exosomal NHE3 was increased in multiple AKI models, including the new and complex AKI model, indicating that NHE3 might serve as a universal AKI marker.

In the I/R-injury AKI rats, the urinary exosomal NHE3 protein was detected at 24 h, as early as the Scr increased. The clamping of renal arteries/veins or pedicles can be performed using two methods: unilaterally or bilaterally. The bilateral clamping of renal arteries, which we performed in the experiments, tends to influence the total renal mass and elevate the Scr and BUN levels within 24 h, which are the characteristic features of AKI in a clinical setting [14]. Our original intention was to search for earlier markers in I/R AKI rats, but the urine output during 0–12 h was quite low due to the anesthesia and operation, and not enough for extracting urinary exosomes or testing the urinary creatinine. Therefore, this study did not focus on early markers in I/R AKI. It still had the practicability and convenience of being able to use urine, avoiding having to take blood frequently.

Due to the limited urine volume in AKI, we did not compare the urinary exosomal NHE3 with other new biomarkers, such as kidney injury molecule-1, N-acetyl-beta-D-glucosaminidase, neutrophil gelatinase-associated lipocalin, liver fatty acid-binding protein, or the urinary products of tissue inhibitor metalloproteinase and insulin growth factor binding protein-7. Another limitation is that we do not present a time course for human urinary exosome NHE3 expression, hence it was not clear whether NHE3 was elevated earlier than Scr in sepsis-associated AKI. Additionally, the mechanism of the increase in exosomal NHE3 in different models of AKI remains to be explored in the future.

The advantage of urinary exosomal NHE3 as a diagnostic marker is that it is elevated in a variety of AKIs, even earlier than Scr in some types of AKI, and collecting urine is noninvasive and easy. The disadvantage is the fact that the extraction of exosomes is quite complicated, requiring ultracentrifugation, and the required assay kits are currently expensive. This problem will be solved with the popularization of exosome extraction and detection technology. In addition, a study in humans with a large sample size is lacking, and is our next research plan.

## 5. Conclusions

Urinary exosomal NHE3 was elevated in various AKI rats and sepsis-associated AKI patients, and increased earlier than Scr in cisplatin-induced AKI and low-NaCl-with-candesartan-related AKI. Urinary exosomal NHE3 may be used as a predictive diagnostic biomarker of AKI in the future.

## Data Availability

The data presented in this study are available on request from the corresponding author.
